# Unilateral Loss of Maxillary Molars in Young Mice Leads to Bilateral Condylar Adaptation and Degenerative Disease

**DOI:** 10.1002/jbm4.10638

**Published:** 2022-07-03

**Authors:** Christopher Phillip Chen, Jiehua Zhang, Bin Zhang, Mohamed G. Hassan, Kyle Hane, Caroline C. Chen, Ana Alejandra Navarro Palacios, Sunil Kapila, Andrew H. Jheon, Alice F. Goodwin

**Affiliations:** ^1^ Division of Craniofacial Anomalies, Department of Orofacial Sciences University of California, San Francisco (UCSF) San Francisco CA USA; ^2^ Program in Craniofacial Biology UCSF San Francisco CA USA; ^3^ Department of Stomatology Renmin Hospital of Wuhan University Wuhan China; ^4^ Hubei Province Key Laboratory of Oral and Maxillofacial Development and Regeneration Wuhan China; ^5^ Department of Oral and Maxillofacial Surgery, Guanghua School of Stomatology, Hospital of Stomatology Sun Yat‐sen University Guangzhou China; ^6^ Guangdong Provincial Key Laboratory of Stomatology Sun Yat‐sen University Guangzhou China; ^7^ Department of Orthodontics, Faculty of Dentistry Assiut University Assiut Egypt; ^8^ Division of Bone and Mineral Diseases, Department of Medicine, School of Medicine Washington University in St. Louis St. Louis MO USA; ^9^ Division of Orthodontics, Department of Orofacial Sciences University of California San Francisco (UCSF) San Francisco CA USA

**Keywords:** CONDYLE, MANDIBLE, MORPHOLOGY, MANDIBULAR CONDYLAR CARTILAGE, MCC, TOOTH EXTRACTION, MALOCCLUSION

## Abstract

The adaptive response of the mandible and temporomandibular joint (TMJ) to altered occlusion in juvenile patients is presently unclear. To address this question, we established a mouse model in which all molars were extracted from the maxillary right quadrant in prepubertal, 3‐week‐old mice and analyzed morphological, tissue, cellular, and molecular changes in the mandible and condyle 3 weeks later. Unilateral loss of maxillary molars led to significant, robust, bilateral changes, primarily in condylar morphology, including anteroposterior narrowing of the condylar head and neck and increased convexity at the condylar surface, as determined by geometric morphometric analysis. Furthermore, both condyles in experimental mice exhibited a degenerative phenotype, which included decreased bone volume and increased mineral density near the condylar head surface compared to control mice. Changes in condylar morphology and mineralized tissue composition were associated with alterations in the cellular architecture of the mandibular condylar cartilage, including increased expression of markers for mature (*Col2a1*) and hypertrophic (*Col10a1*) chondrocytes, suggesting a shift toward differentiating chondrocytes. Our results show significant bilateral condylar morphological changes, alterations in tissue composition, cellular organization, and molecular expression, as well as degenerative disease, in response to the unilateral loss of teeth. Our study provides a relatively simple, tractable mouse tooth extraction system that will be of utility in uncovering the cellular and molecular mechanisms of condylar and mandibular adaptation in response to altered occlusion. © 2022 The Authors. *JBMR Plus* published by Wiley Periodicals LLC on behalf of American Society for Bone and Mineral Research.

## Graphical abstract

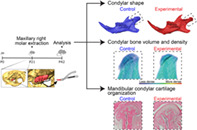

Unilateral extraction of molars in the maxillary right quadrant in 3 week old mice resulted in significant bilateral changes in the mandibular shape, particularly in the condylar process, in experimental mice (red) compared to control (blue) at 6 weeks. Decreased bone volume and increased mineral density were also observed in experimental mice as well as alterations in mandibular condylar cartilage organization compared to control. The unilateral tooth extraction model provides a simple, tractable mouse model to study the mechanisms of condylar adaptation in reponse to altered occlusion.

## Introduction

The temporomandibular joint (TMJ) is a complex structure that includes the articular surface of the temporal bone, joint capsule, articular disc, mandibular condyle, temporomandibular ligament, and lateral pterygoid muscle. TMJ disorders (TMDs) are a group of conditions that involve dysfunction in the jaw joint and associated muscles/ligaments and are often associated with acute/chronic pain which can severely affect quality of life.^(^ ^1^, ^2^, ^3^ ^)^ The prevalence of TMDs is unclear, although 10.6% to 68.1% in males and 21.1% to 72.4% in females has been reported.^(^ ^4^ ^)^ The wide prevalence ranges underscore the difficulty in defining and diagnosing TMDs whose etiology is multifactorial and not fully understood. And if diagnosed with TMD, treatments are largely limited to palliative care or surgery for severe cases, and all have mixed outcomes. To improve upon current diagnostic and treatment methods, a better understanding of the etiopathogenesis of TMDs at the tissue, cellular, and molecular levels is necessary.

Numerous animal models have been developed to induce and study TMDs by altering the four known etiological factors: (i) inflammatory factors such as chemicals, enzymes, and hormones injected in and around the TMJ; (ii) trauma and mechanical factors such as the surgical induction of anterior disc displacement (commonly associated with the beginning of TMD) or disc perforation and prolonged mouth opening; (iii) psychological factors such as stress and anxiety that play pivotal roles in the occurrence and progression of TMDs; and (iv) occlusal factors such as missing or extra teeth that can alter the “bite.”^(^ ^5^ ^)^ Current evidence suggests that altered occlusion, including premature tooth interferences and missing/extra/ectopic teeth are associated with TMDs, but confounding results have impeded clear understanding of the etiopathogenesis of occlusion‐associated TMDs.

Rodent models, specifically anterior crossbite,^(^ ^6^, ^7^, ^8^ ^)^ posterior‐lateral shift,^(^ ^9^, ^10^, ^11^ ^)^ and occlusal interference,^(^ ^12^, ^13^ ^)^ have been utilized to better understand mandibular and condylar adaptation in response to altered occlusion. These models rely on the fabrication and delivery of various appliances to the teeth, which are technically challenging and have been proven to lead to variability between samples. Indeed, studies using various appliances have produced confounding data, such as thickening^(^ ^14^ ^)^ or thinning^(^ ^12^, ^15^ ^)^ of the mandibular condylar cartilage (MCC) in response to altered occlusion. Another approach utilized to model occlusion‐associated TMD, specifically due to tooth loss, is tooth extraction. Unilateral and bilateral tooth extractions have been performed in rhesus monkey,^(^ ^16^ ^)^ sheep,^(^ ^17^ ^)^ rabbit,^(^ ^18^ ^)^ and rat^(^ ^19^, ^20^ ^)^; however, a tooth extraction model, to our knowledge, has surprisingly not yet been developed in mouse, which serves as an economical and easily genetically manipulated model organism.

The mandibular condyle, and specifically the MCC, is unique because it serves a hybrid role as an articular surface as well as a site of rapid growth.^(^ ^21^ ^)^ This rapid growth is made possible by the MCC, a secondary cartilage covering the condyle, which resembles the growth plate in the epiphysis of long bones. However, unlike long bones in which the growth plate eventually mineralizes and disappears, the MCC remains throughout the life of the animal, including in humans.^(^ ^22^ ^)^ The potential and capacity for condylar growth and adaptation in response to occlusal changes are unclear. In humans, remodeling of the condylar subchondral bone^(^ ^23^ ^)^ and MCC^(^ ^24^, ^25^, ^26^ ^)^ has been shown during the progression of TMDs, and many animal studies have shown changes in the condyle and MCC in response to altered occlusion.^(^ ^6^, ^8^, ^18^ ^)^ The superficial layer of the MCC contains fibrocartilage stem cells^(^ ^27^ ^)^ that undergo a proliferative phase, expressing *Col1a1*, *Sox9*, and *Runx2*, differentiate further into maturation stage chondrocytes, expressing *Col2a1*, and then hypertrophic chondrocytes, expressing *Col10a1*. In the deeper layers of the MCC, the tissue is calcified by osteoblasts that migrate into the tissue or transdifferentiate from chondrocytes.^(^ ^28^ ^)^

The aim of this study was to establish a mouse model to reproducibly study the growth and adaptation of the mandibular condyle in response to altered occlusion. Toward this end, we unilaterally extracted upper posterior teeth in rapidly growing mice and characterized the morphological, tissue, cellular, and molecular changes as measures of adaptability, growth, and disease in the mandible and condyle. We extracted teeth at postnatal day 21 (P21) (or prepuberty) and collected 3 weeks later at 6 weeks (postpuberty, sexual maturation) in order to alter occlusion during a significant growth period (ie, prepubertal growth spurt). Three weeks following unilateral extraction of all maxillary right molars in mice at P21, significant bilateral changes in condylar morphology, alterations in bone volume and density, and differences in MCC cellular phenotype, including an increase in mature and hypertrophic chondrocytes, were noted. Additionally, both joints of experimental mice showed increased measures of degenerative disease. Our tooth extraction mouse model provides a simple, tractable, and reproducible model to advance understanding of the adaptive response of the mandible and condyle to altered occlusion.

## Materials and Methods

### Animals

**Fig. 1:**
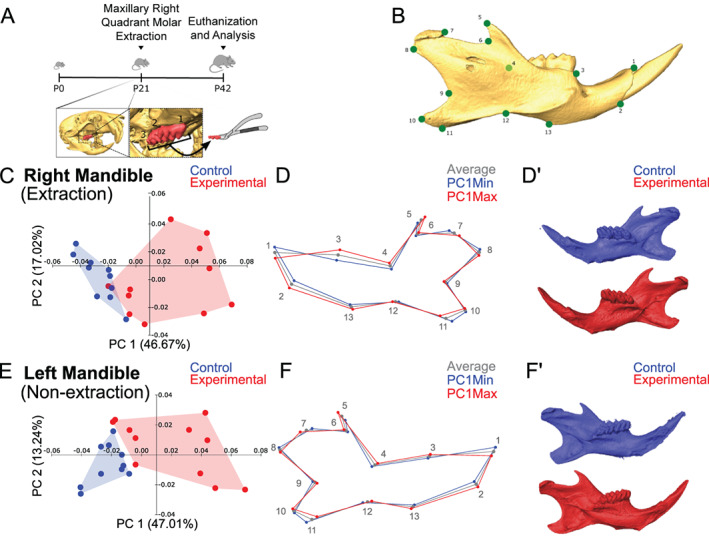
Extraction of maxillary right molars results in significant changes in mandibular shape. (*A*) Schematic of experimental design. All three molars were extracted from the maxillary right quadrant at P21, and mice were euthanized for analysis at P42. (*B*) Isosurface of a hemi‐mandible with landmarks utilized in the study (green dots). (*C*) PCA comparing right extraction mandibles shows that the control (in blue) and experimental (in red) samples separated along PC1 and PC2. (*D*) Wireframes showing average (in gray), PC1 Minimum (Min; in blue), and PC1 Maximum (Max; in red) show increased alveolar bone height (point 3), increased height at the lower border of the mandible (points 2 and 13), decreased length of the coronoid process (point 5), narrowing of the angular process (point 11), and increased posterior‐inferior tip of the condylar process (points 7 and 8) in the right (extraction) mandible in the experimental mice compared to control. (*D′*) Representative isosurfaces of experimental (PC1 max in red) and control (PC1 min in blue) mandibles. PCA (*E*), wireframes (*F*), and isosurfaces (*F′*) of left (non‐extraction) mandibles show similar shape differences in the lower border of the mandible and coronoid, angular, and condylar processes compared to control. *n* = 11 experimental and 10 control mice. P = postnatal day; PCA = principal component analysis.

FVB/NJ mice (The Jackson Laboratory, Bar Harbor, ME, USA) were selected for these experiments as an outbred strain with large litters. Mice were anesthetized with ketamine (80–100 mg/kg) and xylazine (5–10 mg/kg) at P21 (3 weeks of age). Mice were stabilized in a plaster cast, and a mouth prop and cheek retractors were utilized (bent in 0.036″ stainless steel wire) to access the maxillary molars (Supplemental Fig. SS1). All maxillary right molars were surgically removed (right = extraction side) using fine‐tipped tweezers with slightly rounded tips while retaining the maxillary left molars (left = non‐extraction side) in experimental mice (*n* = 17; nine females, eight males for all experiments; Fig. 1*A*). Control mice were exposed to the same conditions (anesthesia and cheek retraction); however, no teeth were extracted (*n* = 17; nine females, eight males collected for all experiments). Food and water were available ad libitum. Mice were housed under a 12‐hours:12‐hours light:dark cycle at a constant temperature of 22 ± 1°C and humidity of 50% ± 5%. The mice were euthanized 3 weeks later at P42 or 6 weeks of age using CO_2_ inhalation and cervical dislocation, and mouse heads were fixed in 4% paraformaldehyde (PFA) for 48 hours at 4°C. All aspects of animal care and experiments were approved by the Institutional Animal Care and Use Committee (IACUC) at UCSF and performed under animal research protocol number AN182286.

### Micro‐computed tomography

Micro‐computed tomography (μCT) was performed on the entire skull of *n* = 11 (five females, six males) experimental and *n* = 10 (four females, six males) control mice using a MicroCT50 (SCANCO Medical AG, Brüttisellen, Switzerland), 55 kVp, 109 μA, 6 W at 20 μm voxel size, with a 500 ms integration time and a 20.5 mm field of view. The scanner was calibrated for bone using an aluminum 0.5 mm filter calibrated to 1200 mg hydroxyapatite (HA)/cm^3^, scaling 4096. Sample numbers were selected based on our power calculation that 11 samples were required to detect a 0.1 mm difference at a power of 90%. We unfortunately lost a control female skull due to fracture during processing, and so *n* = 10 for control.

### Geometric morphometric analysis

**Fig. 2:**
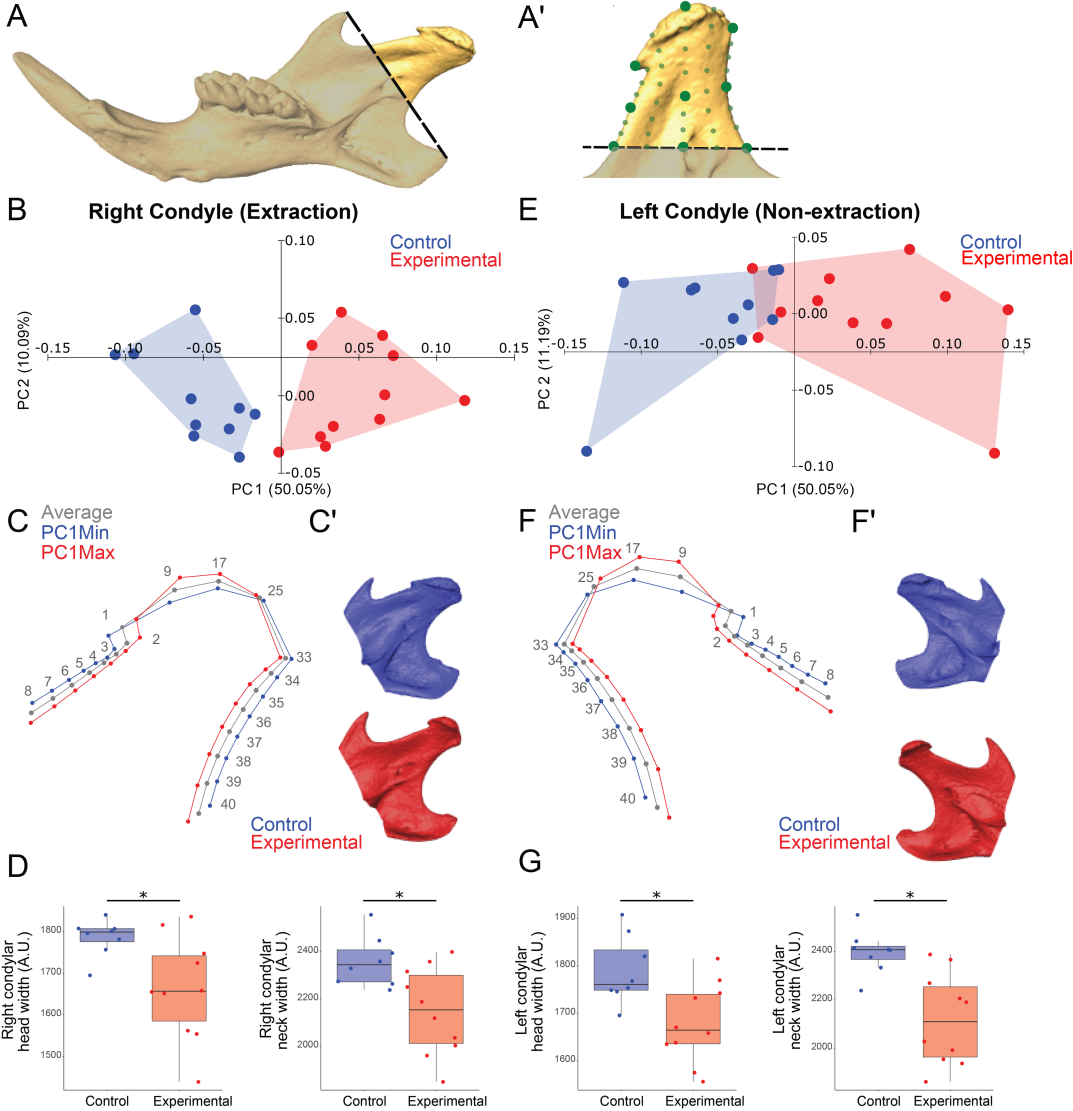
Extraction of maxillary right molars alters the shape of the condylar processes significantly. (*A*) Isosurface of a hemi‐mandible showing region of condylar process measured (demarcated by dashed line connecting the angular and coronoid processes). (*A'*) Isosurface of condyle showing landmarks (large green dots) and semi‐landmarks (small green dots) utilized on the medial surface (40 landmarks). The same number of landmarks were used on the lateral surface (not shown) for a total of 80 landmarks. (*B*) PCA comparing right (extraction) condyles shows that the control (in blue) and experimental (in red) separate along PC1 and PC2. (*C*) Wireframes showing average (in gray), PC1 Min (in blue), and PC1 Max (in red) of right condyles show the extraction condylar head and neck were narrower and condylar surface more convex compared to control. (*C′*) Representative isosurfaces of control and right extraction condyles. (*D*) Linear measurements of right condylar head and neck widths were decreased in experimental compared to control. PCA (*E*), wireframes (*F*), isosurfaces (*F′*), and linear measurements (*G*) show left non‐extraction condyles were also narrower and more convex at the surface in experimental versus control. *n* = 11 experimental and 10 control mice; *p < 0.05. PCA = principal component analysis.

μCT data were imported into Avizo Lite software (version 9.1.1; Thermo Fisher Scientific, Waltham, MA, USA). The cranium and mandibles were segmented using consistent thresholds, and isosurfaces were generated for each anatomical region. The condylar region for bone volume and density measurements and semi‐landmarking was defined by a line between the coronoid and angular processes (Fig. 2*A*,*A'*). Isosurfaces were imported into Landmark software and landmarked. Cranium/midface was landmarked with 44 points (Supplemental Fig. SS2),^(^ ^29^ ^)^ 13 landmarks for the hemi‐mandible (26 for the entire mandible; Fig. 1*B*),^(^ ^30^ ^)^ and 80 sliding semi‐landmarks for each condyle (40 lateral and 40 medial) designed to recapitulate the structure of the condylar processes (Fig. 2*A'*). For semi‐landmarking, each array included nine landmarks that bound the equidistantly placed semi‐landmarks (Fig. 2*A'*), as established by our laboratory.^(^ ^30^, ^31^ ^)^ Intraobserver and interobserver reliability tests were performed to analyze the reproducibility of landmarking (details in Supplemental Materials and Methods).

Landmark coordinates were exported as text files and imported into MorphoJ (V2, Apache License; https://morphometrics.uk/MorphoJ_page.html) for statistical evaluation of shape differences.^(^ ^32^, ^33^ ^)^ Centroid size, defined as the square root of the sum of squared deviations of landmarks from their centroid, was examined between control and experimental groups for each anatomical region using a Student's *t* test; no significant differences were found, and so there was no need to account for size difference in the data (Supplemental Table SS1). To eliminate the effects of orientation, size, and position, Procrustes superimposition was performed on the landmark data. To examine the major differences in shape between the control and experimental groups, principal component analysis (PCA) was conducted. Canonical variate analysis (CVA) or a linear discriminant analysis was also performed for each anatomical region. From this analysis, the Procrustes and Mahalanobis distances among groups were found and permutation tests (10,000 permutations) were conducted to generate *p* values (Supplemental Table SS1). Please find additional information on data representation for all figures in Supplemental Materials and Methods.

### Bone volume and density analysis

To measure bone volume and density of the cranium/midface, mandible, and condyle after segmentation, the volume and total voxel intensity was extracted from Avizo using the Material Statistics package.

### Histological analysis and RNAscope

Six‐week‐old mouse heads fixed in 4% PFA for 2 days were demineralized in 0.5M EDTA for 3 weeks, paraffin processed, embedded coronally, and sectioned at 7 μm (Leica microtome; Leica, Wetzlar, Germany). Hematoxylin and eosin (H&E) and Safranin O staining were performed following standard protocols. RNAscope (Advanced Cell Diagnostics, Newark, CA, USA), an in situ hybridization assay for detection of target RNA within intact cells, was performed following standard manufacturers’ protocols with specific mouse probes against *Col1a1* (catalogue number (Cat No) 319371), *Col2a1* (Cat No 407221), and *Col10a1* (Cat No 426181). Experimental and control mice (*n* = 5 and *n* = 6, respectively) were analyzed for H&E, *n* = 9 experimental and *n* = 9 control for Safranin O, and *n* = 5 experimental and *n* = 4 control mice for RNAscope experiments. Sections were imaged using an Olympus camera (Model DP74‐CU; Olympus, Waltham, MA, USA; SN 9M97049) on a light microscope (Nikon Eclipse E800; Nikon, Tokyo, Japan). The fibrocartilage (FC) and calcified cartilage (CC) widths were measured on H&E‐stained sections. Modified Mankin scoring^(^ ^15^, ^34^ ^)^ was utilized to quantify the cartilage phenotype on Safranin O–stained sections (Supplemental Table S2). RNAscope expression was quantified in Fiji (Image J software, version 2.0.0‐rc‐69/1.52p; NIH, Bethesda, MD, USA; https://imagej.nih.gov/ij/) by selecting the red color in images of sections at consistent thresholds and measuring the pixel area in the MCC.

## Results

### Geometric morphometric analysis reveals bilateral mandibular morphological changes due to unilateral maxillary molar extractions

We observed significant changes in the shape of the mandible in experimental mice with tooth extractions compared to control as determined by geometric morphometric analysis (GMA) (Supplemental Table SS1). When we compared the entire mandibles (right and left hemi‐mandibles together) of experimental and control samples, we found the control and experimental mandibles clustered distinctly by shape (Supplemental Fig. SS3). Principal component (PC) 1 and PC2 were associated with 41.8% and 13.4% of the total shape variation observed, respectively (Supplemental Fig. SS3 *A*). These data suggest the right and left hemi‐mandibles of the experimental mice were more similar in shape to each other than control.

To further assess the mandibular shape differences in experimental mice compared to control, we compared the right hemi‐mandibles (the side with tooth extraction) in experimental and control and the left (non‐extraction side) hemi‐mandibles separately (Fig. 1). Analysis of the right/extraction side hemi‐mandibles showed distinct separation of clusters with PC1 and PC2 associated with 46.67% and 17.02% of the total variance, respectively (Fig. 1*C*). Similarly, the left/non‐extraction side hemi‐mandibles showed distinct clusters with PC1 and PC2 associated with 47.01% and 13.24% of the total variation, respectively (Fig. 1*E*). Wire frames of the hemi‐mandibles showing the position of each landmark for PC1 Maximum (Max), PC1 Minimum (Min), and the average (Fig. 1*D*,*F*) as well as isosurfaces of the hemi‐mandibles of specimens representative of PC1 Max and PC1 Min (Fig. 1*D'*,*F′*) show the mandibular morphology differences observed. Significant shape differences were noted with experimental hemi‐mandibles showing increased height at the lower border of the mandible (captured by points 2 and 13 in Fig. 1*D*,*F*), decreased length of the coronoid process (point 5), narrowing of the angular process (point 11), and increased posterior‐inferior tip of the condylar process (points 7 and 8) on both the right/extraction and left/non‐extraction sides compared to control. Overall, the shape of the right and left hemi‐mandibles in the experimental mice was similar with the exception of increased mandibular alveolar height due to the over‐eruption of mandibular molars on the right/extraction side (Fig. 1*D*, point 3). Linear measurements also verified the difference in alveolar height (Supplemental Fig. SS4). Additionally, CVA was performed and confirmed distinct clustering between control and experimental hemi‐mandibles (Supplemental Fig. SS5).

### GMA reveals robust, bilateral shape changes in the mandibular condyles

To measure specific shape changes in the mandibular condyles (head and neck), we included landmarks and semi‐landmarks on the condylar process (Fig. 2*A*,*A'*) and performed GMA.^(^ ^30^, ^31^ ^)^ Semi‐landmarking analysis revealed an increase in convexity at the condylar surface and a narrowing of the condylar head and neck in both the anteroposterior and mediolateral dimensions in experimental condyles. Specifically, control and experimental right/extraction side condyles clustered distinctly along PC1 (50.05%) and PC2 (10.09%; Fig. 2*B*). The experimental extraction side condyles were narrower in the anteroposterior dimension, and the head surface was more convex compared to control (Fig. 2*C*,*C'*). Similarly, experimental left/non‐extraction side condyles significantly separated from control along PC1 (50.05%) and PC2 (11.19%), albeit with increased variability (Fig. 2*E*). Despite this increased variability, similar shape changes were observed between the left/non‐extraction side and right/extraction side in the experimental mice, including narrowed anteroposterior dimensions and increased convexity of the head surface (Fig. 2*F*,*F'*). Moreover, linear measurements confirmed significant and similar narrowing of the right/extraction and left/non‐extraction side condylar head and neck widths in experimental compared to control (Fig. 2*D*,*G*).

### GMA reveals subtle shape changes in the cranium/midface

The cranium/midface, which houses the cranial vault, cranial base, and maxilla, showed significant asymmetric growth and shape differences between control and experimental mice (Supplemental Table SS1). However, these differences were primarily in the alveolar process length (distance between most mesial point of the first molar (points 30 and 31) and the most distal point of the third molar (points 32 and 33; Supplemental Fig. SS2 *A'*). Unfortunately, these points were difficult to reliably landmark in the maxillary right quadrant of the experimental mice in which the molars were extracted, and thus, the biological significance of this finding is unclear. No other landmarking points showed significant change in experimental skulls compared to control. Notably, PCA and CVA showed no sex‐specific differences between control and experimental skulls (cranium/midface and mandible; Supplemental Fig. SS6). Thus, both male and female mice were used for analyses.

### Decreased bone volume and increased bone density in experimental condylar processes

**Fig. 3:**
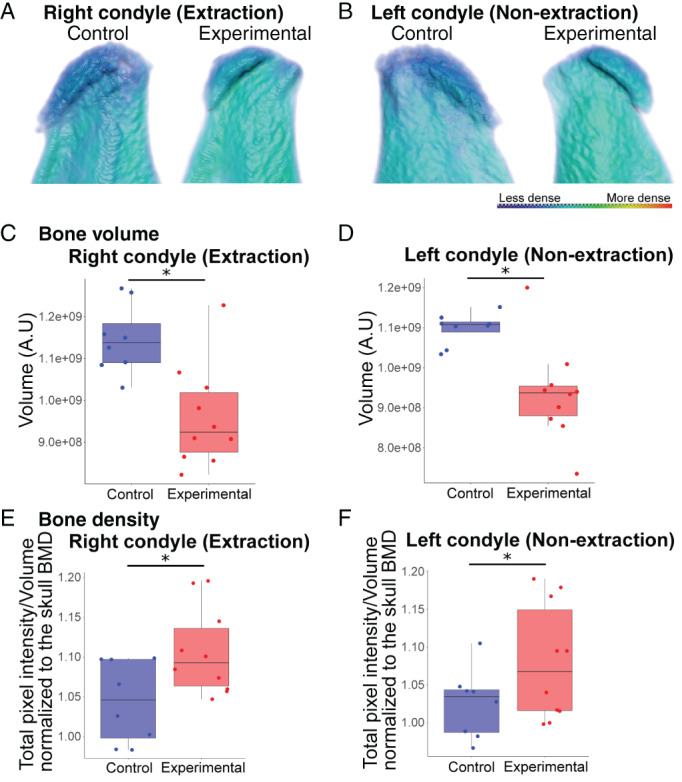
The condylar processes in extraction mice have decreased bone volume and increased bone mineral density compared to control. (*A*) Renderings of the condylar processes with the color representing the relative bone mineral density (scale: red, more dense and blue, less dense) show that the experimental condylar processes had decreased bone volume and increased bone density, particularly near the condylar head surface, compared to control on the right (extraction) side. (*B*,*C*) Quantification shows a significant decrease in bone volume (*B*) of the right (extraction) condylar processes in the experimental samples compared to control and significant increase in bone mineral density (*C*). There was a decrease in bone volume and increase in relative bone mineral density of the experimental condylar processes in the experimental compared to control on the left (non‐extraction) side as shown in the renderings (*D*) and quantified in the graphs of bone volume (*E*) and bone density (*F*). *n* = 11 experimental and 10 control mice; *p < 0.05.

We determined that the total bone volume of experimental extraction and non‐extraction condylar processes was decreased by 14.86% and 16.17%, respectively, compared to control (Fig. 3*A*–*D*). We then sought to determine whether the bone quality was similar by assessing the bone mineral density (BMD). Relative BMD was significantly increased 4.96% and 5.60% in the experimental extraction and non‐extraction condylar processes, respectively, compared to control (Fig. 3*E*,*F*). The increase in BMD appears to be primarily localized to the condylar head (Fig. 3*A*,*B*). Thus, total bone volume decreased, whereas BMD increased in the experimental condylar processes near the head surface. There were no significant BMD differences between experimental and control mice in the remaining skull (data not shown). Thus, alterations in BMD were specific to the condylar processes.

**Fig. 4:**
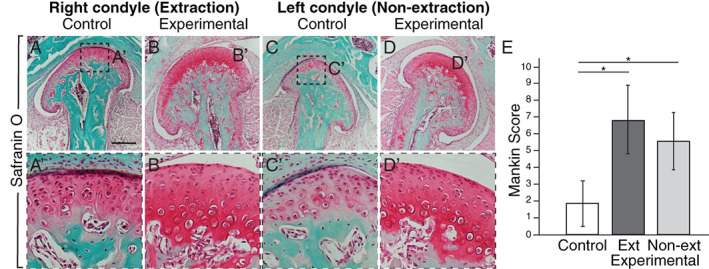
The condylar processes in extraction mice have osteoarthritic‐like changes. (*A*–*D′*) Safranin O staining shows increased staining in the experimental mice on the right (extraction *B*,*B′*) and left (non‐extraction *D*,*D′*) sides compared to control (*A*,*A'*,*C*,*C′*). (*E*) Mankin scoring was performed (Supplemental Table S2 describes scoring criteria), and the scores for the Ext and Non‐ext experimental condyles were significantly increased compared to control. *n* = 9 experimental and 9 control mice; *p < 0.05; scale bar = 200 μm. Ext = extraction; Non‐ext = non‐extraction.

The alterations in condylar bone were accompanied by cellular changes suggesting an osteoarthritic phenotype in experimental mice compared to control as indicated by increased pericellular and background staining, clustering of cells, and erosion of the cartilage surface in the experimental condyles (Fig. 4*A*–*D'*, Supplemental Table S2). Quantitatively, these changes contributed to significantly increased Mankin scores in both condyles of the experimental mice compared to control (Fig. 4*E*), indicative of osteoarthritic, degenerative changes in the experimental condylar cartilage.

### Increase in maturation stage and hypertrophic chondrocytes in the mandibular condylar cartilage of experimental mice

**Fig. 5:**
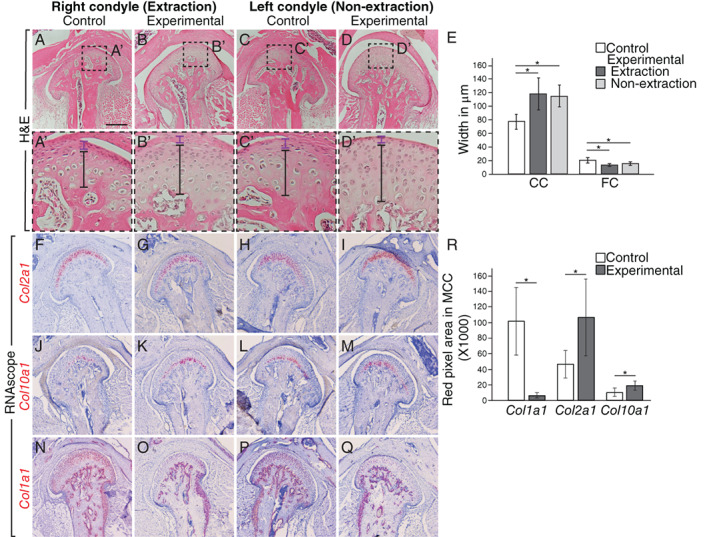
Extraction results in alterations in the cellular and molecular structure of the mandibular condylar cartilage with an increase in maturation and hypertrophic chondrocytes (*A*–*D′*) H&E staining shows the CC layer (demarcated by black line) of the MCC was thicker and the FC layer (marked by purple line) was thinner in the experimental compared to control in the right (extraction; *A*–*B′*) and left (non‐extraction; *C*–*D′*) condyles. (*E*) Graph showing quantification of the CC and FC widths in μm in the control right and left sides (white bar), experimental right/extraction side (dark gray), and experimental left/non‐extraction side (light gray). CC was significantly increased and FC was significantly decreased in extraction and non‐extraction experimental condyles compared to control. (*F*–*Q*) RNAscope with probe against *Col2a1* (red, counterstain purple) shows increased expression in both the left/non‐extraction (*I*) and right/extraction (*G*) side experimental MCC compared to control (*F*,*H*). There was increased expression of *Col10a1* in the MCC of non‐extraction (*K*) and extraction (*M*) condyles in experimental mice compared to control (*J*,*L*). *Col1a1* expression in the subchondral bone was similar, however, *Col1a1* was decreased in the superficial layer of the MCC in the experimental condyles (*O*,*Q*) compared to control (*N*,*P*). (*R*) Graph of quantification of the RNAscope expression. *n* = 5 experimental and 6 control mice for H&E and *n* = 5 experimental and 4 control mice for RNAscope; *p < 0.05; scale bar = 200 μm. CC = calcified cartilage; FC = fibrocartilage; MCC = mandibular condylar cartilage.

To further investigate the basis for the adaptive and degenerative changes in the experimental condyles at the cellular and molecular level, TMJ condyle histomorphometry was performed. The fibrocartilage (FC) layer, including the superficial fibrous and proliferative chondrocytes, and the calcified cartilage (CC) layer, composed of mature and hypertrophic chondrocytes embedded in extracellular matrix (ECM), were measured. These regions were measured as they had clearly defined boundaries based on cellular morphology.^(^ ^35^ ^)^ The width of the FC was significantly decreased and the CC was significantly increased in the experimental right/extraction and left/non‐extraction condyles compared to control (Fig. 5*A*–*E*).

Considering the changes in the MCC zonal architecture of experimental condyles, we assayed for levels of chondrocyte markers *Col2a1* and *Col10a1* and osteoblast/fibroblast marker *Col1a1* to determine MCC composition at the molecular level. *Col2a1* (Fig. 5*F*–*I*) and *Col10a1* (Fig. 5*J*–*M*) expression was increased in experimental extraction and non‐extraction condyles compared to control (Fig. 5*R*). Thus, the increase in marker expression and expansion of the cell regions suggests there was expansion of maturation and hypertrophic chondrocytes in response to altered occlusion. *Col1a1* expression was unchanged in control and experimental condylar subchondral bone (data not shown) but was decreased in experimental MCCs compared to control (Fig. 5*N*–*R*). Overall, our data show that the unilateral loss of maxillary molars resulted in a shift toward further differentiated chondrocytes in the MCC.

## Discussion

To test the effects of asymmetric tooth loss and subsequent altered occlusion on craniofacial skeletal adaptation and disease in rapidly growing animals, we unilaterally extracted all molars from the maxillary right quadrant in P21 mice and analyzed the skulls at P42. We discovered that unilateral tooth loss resulted in robust, bilateral shape changes in experimental (extraction and non‐extraction side) mandibles, most strikingly in the condyles. Both left and right experimental condyles were narrower and showed increased convexity of the condylar head surface. Our studies are the first to exploit GMA to catalog three‐dimensional (3D) morphologic changes in the mandibular condyles.^(^ ^30^, ^31^ ^)^ Furthermore, both experimental condylar processes showed an ~15% decrease in total bone volume but an ~5% increase in BMD compared to control condyles. Increased degenerative changes were noted in both left and right condyles from experimental mice, and the proliferative and differentiated cartilage zones within the MCC were altered with a shift toward markers of maturation and hypertrophic chondrocytes.

Our tooth extraction mouse model provides important advantages to prior occlusion‐altered animal models. First, prior rodent models with anterior crossbites,^(^ ^6^, ^7^, ^8^ ^)^ posterior‐lateral shifts,^(^ ^9^, ^10^, ^11^ ^)^ or occlusal interferences,^(^ ^12^, ^13^ ^)^ are all “additive” models in that appliances are bonded to induce specific changes in occlusion. Unfortunately, these “additive” models are heavily dependent on appliance design/fabrication and operator technique that are difficult to reproduce. This fact is evidenced in studies using appliances to induce similar occlusal changes, such as disclusion of the teeth with buildups/appliances increasing the height of the molars or incisors, only to generate opposing, confounding data, such as thickening^(^ ^14^ ^)^ or thinning^(^ ^12^, ^15^ ^)^ of the MCC. Conversely, tooth extractions are “subtractive,” relatively simple, tractable, and do not need subsequent appliance checks. Second, unilateral and bilateral tooth extractions to induce changes in the TMJ have been performed on rhesus monkey,^(^ ^16^ ^)^ sheep,^(^ ^17^ ^)^ rabbit,^(^ ^18^ ^)^ and rat,^(^ ^19^, ^20^ ^)^ but surprisingly, not in mice. The relatively small size of mice, especially at P21, may have previously precluded their utilization. We demonstrate here that mouse molars can be extracted with ease to induce changes in the condyles. Moreover, we will take advantage of the numerous transgenic mouse lines with specific gene activations or altered signaling pathways for in vivo mechanistic studies. Third, we can readily perform tooth extractions in post‐P21 mice to determine the potential and capacity for growth, adaptation, and degeneration in the mandibular condyle as mice age.

The MCC has growth and adaptive potential, as shown by alterations in the MCC during progression of TMJ osteoarthritis in humans^(^ ^24^, ^25^, ^26^ ^)^ and in response to altered occlusion in animal models^(^ ^6^, ^8^, ^18^ ^)^; however, the extent of the adaptive potential and the mechanisms underlying it are not understood. In our study, there was an increase in the thickness of the MCC, particularly in the CC layer. Notably, Jung and colleagues^(^ ^12^ ^)^ observed a decrease in MCC thickness when mouse maxillary left molars were built up with resin to produce premature occlusal contact. The increased thickness in the MCC was accompanied by increased expression of mature (*Col2a1*) and hypertrophic (*Col10a1*) chondrocyte molecular markers. These data show a shift toward differentiated chondrocytes as part of the adaptive response in the MCC. Alterations in MCC cellular composition^(^ ^18^ ^)^ and expression of collagen proteins^(^ ^14^ ^)^ have been shown in animal models; however, here we rely on cellular morphology and molecular markers of chondrocytes to clearly show the response of the chondrocytes in the MCC to asymmetric occlusion. These chondrocytes may then transdifferentiate into osteoblasts, as has been shown,^(^ ^36^, ^37^ ^)^ and remodel bone, generating the changes in condylar shape and BMD observed. Lineage tracing experiments in our tooth extraction model will provide more insight into the fate of the MCC cells during the adaptive response.

The decreased bone volume and increased Mankin scores in the experimental condylar processes compared to control suggest that an osteoarthritis‐like phenotype was induced with unilateral tooth extractions. Although intermediate stages of change were not assessed, it is possible that there may have been initial bone loss and degeneration in the first 1 to 2 weeks after extraction due to decreased function, followed by initiation of an adaptive response, as suggested by the increased density of the bone at the condylar surface in experimental mice compared to control and alterations in the MCC. We will analyze our tooth extraction model at additional stages to better understand the progression of occlusion‐associated TMD.

Improved understanding of how the MCC develops, is maintained, adapts and/or degenerates at the cellular and molecular level will help identify therapeutic targets in growth regulation and inhibition of degeneration of the mandibular condyle in patients. There is tremendous potential with increased understanding of the MCC to improve treatment for skeletal malocclusion, which often includes too much or too little growth of the condyle, and TMJ degenerative disease, in which improved adaptation of the MCC may arrest disease progression. Here, we show the response of the mandibular condyle and MCC to altered occlusion in a tooth extraction mouse model, which may be utilized to further our mechanistic understanding of these adaptive and degenerative processes.

## Author Contributions

**Christopher Phillip Chen:** Data curation; formal analysis; investigation; methodology; validation; visualization; writing – original draft; writing – review and editing. **Jiehua Zhang:** Data curation; formal analysis; investigation; methodology; validation; visualization; writing – original draft; writing – review and editing. **Bin Zhang:** Data curation; formal analysis; investigation; validation; writing – review and editing. **Mohamed G. Hassan:** Data curation; formal analysis; investigation; methodology; validation; visualization; writing – review and editing. **Kyle Hane:** Data curation; formal analysis; methodology; validation; writing – review and editing. **Caroline C. Chen:** Data curation; formal analysis; investigation; methodology; validation; visualization; writing – review and editing. **Ana Alejandra Navarro Palacios:** Conceptualization; investigation; methodology; writing – review and editing. **Sunil Kapila:** Investigation; methodology; writing – review and editing. **Andrew H. Jheon:** Conceptualization; data curation; funding acquisition; investigation; methodology; project administration; resources; supervision; validation; visualization; writing – original draft; writing – review and editing. **Alice F. Goodwin:** Conceptualization; data curation; formal analysis; funding acquisition; investigation; methodology; project administration; resources; supervision; validation; visualization; writing – original draft; writing – review and editing.

## Conflict of Interest

The authors declare no potential conflicts of interest with respect to the authorship and/or publication of this article.

### Peer Review

The peer review history for this article is available at https://publons.com/publon/10.1002/jbm4.10638.

## Supporting information


**Supplemental Fig. S1**. Experimental set up for tooth extractions. Mouse was placed in a stabilizing holder and a mouth prop and cheek retractors were used to access the right maxillary molar teeth for extraction.

**Supplemental Fig. S2**. Cranium landmarks. (*A*) Dorsal and (*A'*) ventral views of the isosufaces of the cranium with landmarks utilized for the study marked by numbered green dots.

**Supplemental Fig. S3**. Unilateral molar extraction results in significant bilateral mandibular shape changes. (*A*) Principal component analysis (PCA) comparing both right and left mandibles shows that the control (in blue) and experimental (in red) samples separated along PC1 and PC2. (*B*) Wireframes showing average (in gray), PC1 Minimum (Min; in blue), and PC1 Maximum (Max; in red) of right (solid line) and left (dashed line) hemi‐mandibles. (*B′*) Representative isosurfaces of control (PC1 Min) and experimental (PC1 Max) mandibles.

**Supplemental Fig. S4**. The alveolar height of the right mandible in the extraction mice is significantly increased compared to control. (*A*) Linear measurements of the alveolar height in the right extraction mandible showed significant increase in the experimental mice compared to control (*p < 0.003). (*B*) There was no significant difference in alveolar height in the left mandible between control and extraction mice.

**Supplemental Fig. S5**. Canonical variate analysis on mandibles and condyles in extraction mice compared to control. (*A*–*C*) Canonical variate analysis shows clear separation between both mandibles (*A*), the right/extraction mandible (*B*), the left/non‐extraction mandible (*C*), the right/extraction condyle (*D*), and the left/non‐extraction condyle (*E*) in control (blue) and experimental (red) samples.

**Supplemental Fig. S6**. No clear sex differences are observed in control or experimental samples. (*A*) Principal component analysis (PCA) showed the male and female samples did not cluster in the control or experimental groups, suggesting the shape differences observed due to tooth extraction did not differ significantly between males and females.

Supplemental Materials and Methods

**Supplemental Table S1**. Probability values from statistical hypothesis tests using calculated centroid size and Procrustes distance. Bold indicates *p* < 0.05.
**Supplemental Table S2**. Modified Mankin scoring system utilized.
