# Supplementary figures and images for: Unilateral Loss of Maxillary Molars in Young Mice Leads to Bilateral Condylar Adaptation and Degenerative Disease
Source: JBMR Plus. 2022 Jul 3;6(7):e10638. doi: 10.1002/jbm4.10638 (PMC9289985; doi:10.1002/jbm4.10638)

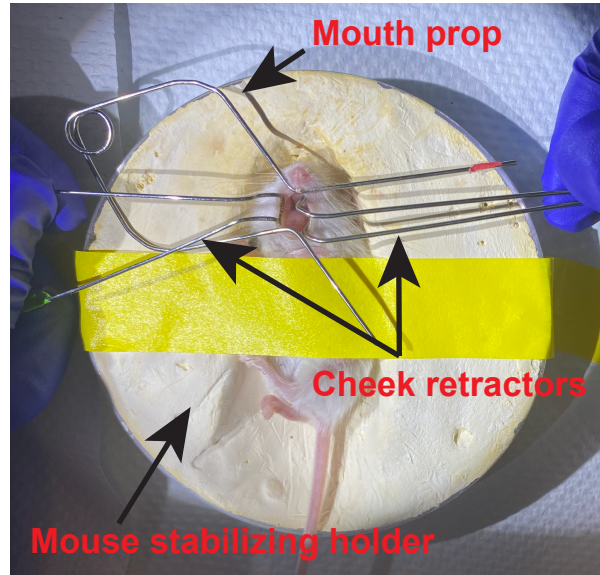

Supplement: Supplementary file 1 — Supplemental Fig. S1. Experimental set up for tooth extractions. Mouse was placed in a stabilizing holder and a mouth prop and cheek retractors were used to access the right maxillary molar teeth for extraction. [file JBM4-6-e10638-s005.pdf]

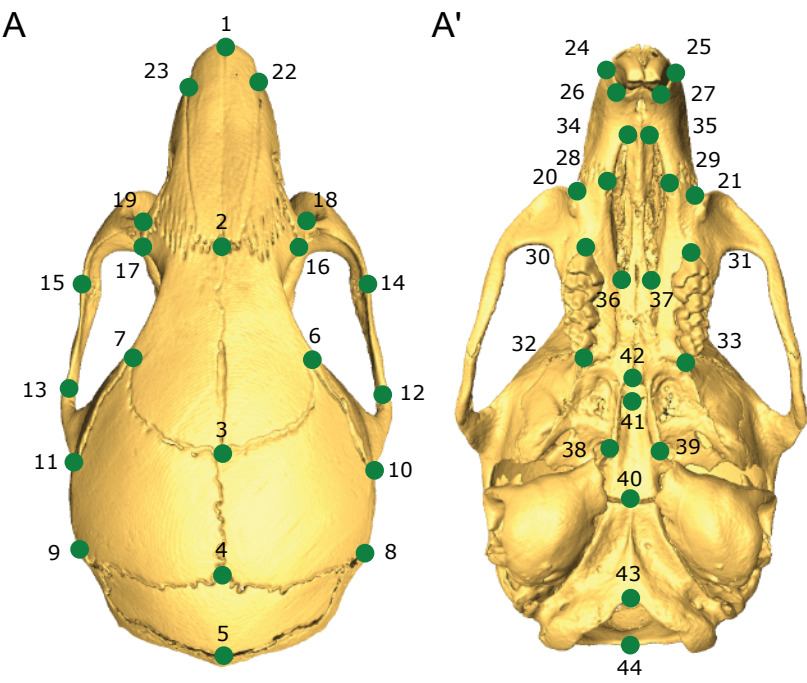

Supplement: Supplementary file 2 — Supplemental Fig. S2. Cranium landmarks. (A) Dorsal and (A') ventral views of the isosufaces of the cranium with landmarks utilized for the study marked by numbered green dots. [file JBM4-6-e10638-s003.pdf]

**A Both Mandibles**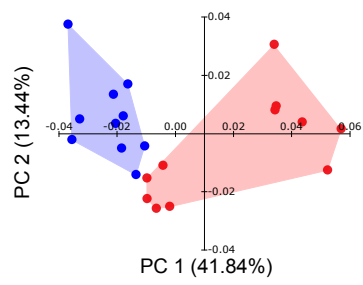**B**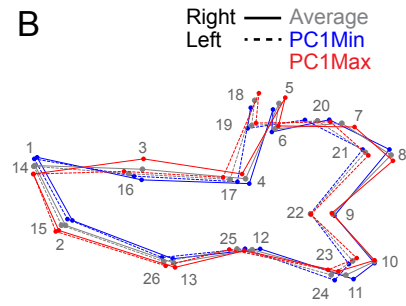**B'**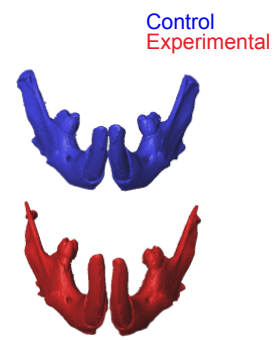

Supplement: Supplementary file 3 — Supplemental Fig. S3. Unilateral molar extraction results in significant bilateral mandibular shape changes. (A) Principal component analysis (PCA) comparing both right and left mandibles shows that the control (in blue) and experimental (in red) samples separated along PC1 and PC2. (B) Wireframes showing average (in gray), PC1 Minimum (Min; in blue), and PC1 Maximum (Max; in red) of right (solid line) and left (dashed line) hemi‐mandibles. (B′) Representative isosurfaces of control (PC1 Min) and experimental (PC1 Max) mandibles. [file JBM4-6-e10638-s001.pdf]

**A Right Mandible (Extraction)**

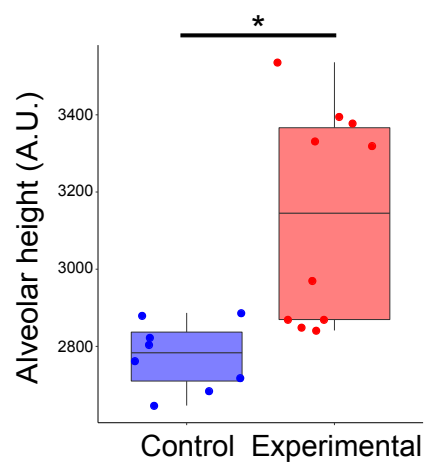

**B Left Mandible (Non-extraction)**

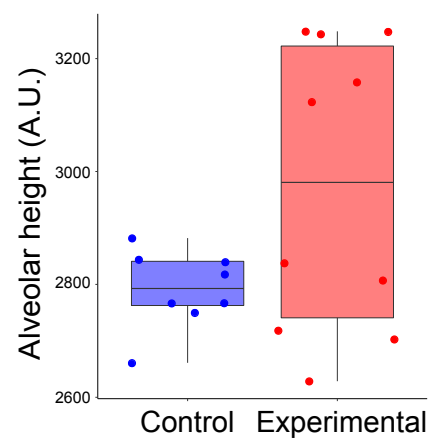

Supplement: Supplementary file 4 — Supplemental Fig. S4. The alveolar height of the right mandible in the extraction mice is significantly increased compared to control. (A) Linear measurements of the alveolar height in the right extraction mandible showed significant increase in the experimental mice compared to control (*p < 0.003). (B) There was no significant difference in alveolar height in the left mandible between control and extraction mice. [file JBM4-6-e10638-s008.pdf]

A

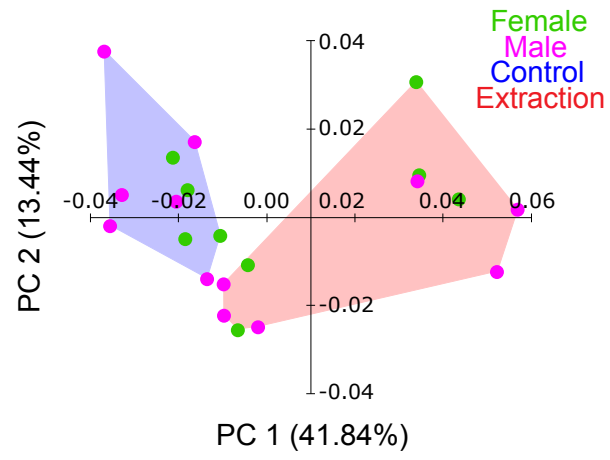

Supplement: Supplementary file 6 — Supplemental Fig. S6. No clear sex differences are observed in control or experimental samples. (A) Principal component analysis (PCA) showed the male and female samples did not cluster in the control or experimental groups, suggesting the shape differences observed due to tooth extraction did not differ significantly between males and females. [file JBM4-6-e10638-s004.pdf]
