# Supplementary material for: Unilateral Loss of Maxillary Molars in Young Mice Leads to Bilateral Condylar Adaptation and Degenerative Disease
Source: JBMR Plus. 2022 Jul 3;6(7):e10638. doi: 10.1002/jbm4.10638 (PMC9289985; doi:10.1002/jbm4.10638)

Supplemental Figure 5.

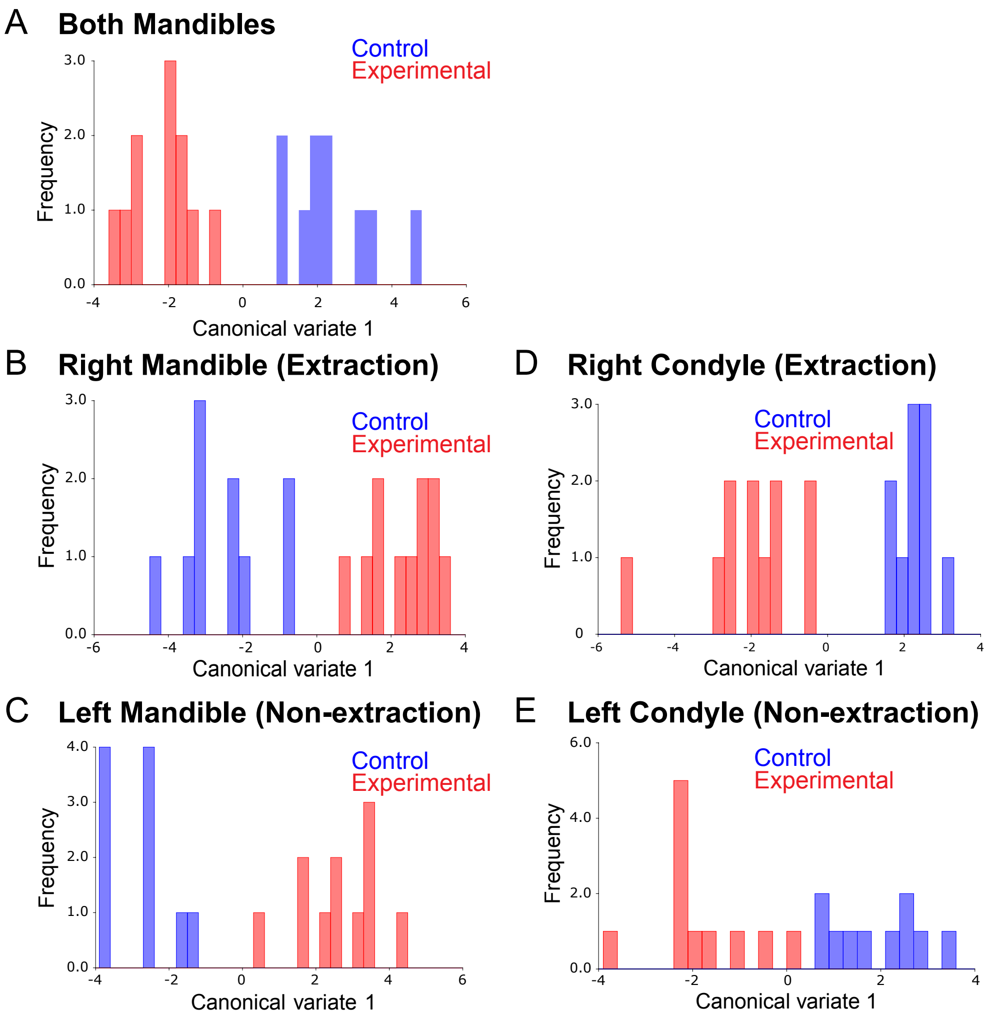

Supplement: Supplementary file 5 — Supplemental Fig. S5. Canonical variate analysis on mandibles and condyles in extraction mice compared to control. (A–C) Canonical variate analysis shows clear separation between both mandibles (A), the right/extraction mandible (B), the left/non‐extraction mandible (C), the right/extraction condyle (D), and the left/non‐extraction condyle (E) in control (blue) and experimental (red) samples. [file JBM4-6-e10638-s007.pdf]
