# Supplementary material for: Unilateral Loss of Maxillary Molars in Young Mice Leads to Bilateral Condylar Adaptation and Degenerative Disease
Source: JBMR Plus. 2022 Jul 3;6(7):e10638. doi: 10.1002/jbm4.10638 (PMC9289985; doi:10.1002/jbm4.10638)
Supplement: Supplementary file 7 — Supplemental Materials and Methods [file JBM4-6-e10638-s002.docx]

**Supplemental Materials and Methods**

*Landmarking intra- and inter-observer reliability tests*

Intra- and inter-observer reliability tests were completed for the landmarking data within and between individuals. Briefly, to determine the accuracy and reproducibility of landmark identification, an initial random subset of 7 samples was landmarked twice using the 44 skull landmarks. We calculated the difference in the matching landmark coordinates from the two measurements (i.e., intra-observer error) and removed those that consistently exceeded an arbitrary difference of 7 voxels (0.125mm) between measurements.[^(1)^](https://sciwheel.com/work/citation?ids=8507773&pre=&suf=&sa=0&dbf=0) Furthermore, we used centroid sizes, the square root of the sum of squared Euclidean distances from each landmark to their own centroid, as a proxy for cranial size.[^(2)^](https://sciwheel.com/work/citation?ids=10018120&pre=&suf=&sa=0&dbf=0) To determine inter-observer landmark reproducibility, two observers (C.P.C. and M.G.H.) independently located the landmarks on 10 randomly selected samples.[^(3)^](https://sciwheel.com/work/citation?ids=9288065&pre=&suf=&sa=0&dbf=0) A 10,000 round permutation test was performed on the Procrustes distance between the observers’ landmarked samples, testing for mean overall shape differences between them.

*Data representation*

The data in Figures 2 and 3 and Supplemental Figure 4 are represented by box and whisker plots; the center line shows the median, and the top and bottom of the boxes represent the 75^th^ and 25^th^ percentile, respectively. The top and bottom whiskers extend the entire range of datapoints except in the case of outliers, which are defined as being equal to the median +/- 1.58 * (interquartile range)/sqrt(n). Error bars in graphs in Figures 4 and 5 represent standard deviation. P-values are described in the figure legends.

References for Supplemental Materials and Methods

[1.    Maga AM, Navarro N, Cunningham ML, Cox TC. Quantitative trait loci affecting the 3D skull shape and size in mouse and prioritization of candidate genes in-silico. Front. Physiol. 2015 Mar 26;6:92.](https://sciwheel.com/work/bibliography/8507773)

[2.    Dryden IL, Mardia KV. Statistical Shape Analysis. 1st ed. Chichester: Wiley; 1998. p. 376.](https://sciwheel.com/work/bibliography/10018120)

[3.    Hassan MG, Kaler H, Zhang B, Cox TC, Young N, Jheon AH. Effects of Multi-Generational Soft Diet Consumption on Mouse Craniofacial Morphology. Front. Physiol. 2020 Jul 10;11:783.](https://sciwheel.com/work/bibliography/9288065)
