# Supplementary material for: Unilateral Loss of Maxillary Molars in Young Mice Leads to Bilateral Condylar Adaptation and Degenerative Disease
Source: JBMR Plus. 2022 Jul 3;6(7):e10638. doi: 10.1002/jbm4.10638 (PMC9289985; doi:10.1002/jbm4.10638)
Supplement: Supplementary file 8 — Supplemental Table S1. Probability values from statistical hypothesis tests using calculated centroid size and Procrustes distance. Bold indicates p < 0.05. Supplemental Table S2. Modified Mankin scoring system utilized. [file JBM4-6-e10638-s006.docx]

**Supplemental Table 1.**

|  | **p-value** | | |
| --- | --- | --- | --- |
|  | **Centroid size** | **Procrustes Distance** | |
|  | **Control/Experiment** | **Control/Experiment** | **Male/Female** |
| **Both Mandibles** | 0.32748866 | **<.0001** | 0.4762 |
| **Right Mandible** | 0.268456023 | **<.0001** | 0.4609 |
| **Left Mandible** | 0.205705201 | **0.0006** | 0.3319 |
| **Right Condyle** | 0.865153994 | **0.0001** | 0.3548 |
| **Left Condyle** | 0.98617899 | **0.0001** | 0.382 |
| **Cranium** | 0.142468966 | **0.0023** | 0.2883 |

**Supplemental Table 1.** Probability values from statistical hypothesis tests using calculated centroid size and Procrustes distance. Bold indicates p<0.05.

**Supplemental Table 2.**

| **Modified Mankin Score Parameter** | **Score** |
| --- | --- |
| **Pericellular Safranin O staining** |  |
| Normal | 0 |
| Slightly enhanced | 1 |
| Intensely enhanced | 2 |
| **Background Safranin O staining** |  |
| Normal | 0 |
| Slightly increased or decreased | 1 |
| Severely increased or decreased | 2 |
| No staining | 3 |
| **Arrangement of chondrocytes** |  |
| Normal | 0 |
| Appearance of clustering | 1 |
| Hypocellularity | 2 |
| **Cartilage erosion** |  |
| Smooth non-eroded cartilage | 0 |
| Rough non-eroded cartilage | 1 |
| Superficial fibrillation | 2 |
| Separation of uncalcified from calcified cartilage | 3 |
| Erosion of uncalcified cartilage only | 4 |
| Erosion extending into calcified cartilage | 5 |
| Erosion extending into subchondral bone | 6 |

**Supplemental Table 2.** Modified Mankin scoring system utilized.
